# Multiple recombinant events in human T-cell Leukemia virus Type 1: complete sequences of recombinant African strains

**DOI:** 10.1080/22221751.2020.1752117

**Published:** 2020-05-13

**Authors:** Olivier Cassar, Alexandra Desrames, Ambroise Marçais, Olivier Gout, Graham P. Taylor, Olivier Hermine, Vicente Soriano, Carmen de Mendoza, Océane Dehan, Margot Le Mener, Philippe V. Afonso, Antoine Gessain

**Affiliations:** a Unité d’Epidémiologie et Physiopathologie des Virus Oncogènes, Institut Pasteur, UMR3569 CNRS, Université de Paris, Paris, France; b Service d’Hématologie, Hôpital Necker-Enfants Malades, Paris, France; c Département de Neurologie, Fondation Rothschild, Paris, France; d Department of Infectious Disease, Imperial College, London, United Kingdom; e UNIR Health Sciences School and Medical Center, Madrid, Spain; f Internal Medicine Laboratory Puerta de Hierro Research Institute, University Hospital Majadahonda, Madrid, Spain

**Keywords:** HTLV-1, molecular epidemiology, recombination, Africa, HTLV-1 complete genome, reverse transcription

## Abstract

Africa is the largest endemic area for HTLV-1, with many molecular genotypes. We previously demonstrated that some strains from North Africa (a-NA clade) originated from a recombinant event between Senegalese and West African strains. A series of 52 new HTLV-1 strains from 13 North and West African countries were sequenced in the LTR region and/or a env gene fragment. Four samples from French Guyanese of African origin were also added. Furthermore, 7 complete sequences from different genotypes were characterized. Phylogenetic analyses showed that most of the new African strains belong to the Cosmopolitan a-genotype. Ten new strains from the a-NA clade were found in Morocco, Western Sahara, Mali, Guinea, Côte d'Ivoire and Ghana. A new a-G-Rec clade, which arose from a distinct recombination event between Senegalese and West African strains, was identified in Guinea and Ghana. The complete sequences suggest that recombination occur in the LTR as well as the env/pol region of the genome, thus a-NA and a-G-Rec strains have a mosaic profile with genetic segments from either a-WA or a-Sen strains. Our work demonstrates that recombination in HTLV-1 may not be as rare an event as previously proposed.

## Introduction

From its discovery by Poiesz *et al.* in 1980 in the USA [1], HTLV-1 has been reported in many countries [2]. Its geographical distribution is characterized by significant heterogeneity, usually with clusters of endemic foci located nearby areas with low to no prevalence. The main high HTLV-1 endemic areas are the Southern part of Japan, West and Central Africa, the Caribbean basin, South America, and some regions of the Middle East and of Australo-Melanesia. Such a distribution is likely related to founder effects in the different populations, followed by virus spread over time [2,3]. While the majority of people living with HTLV-1 infection remain asymptomatic, HTLV-1 has been defined as the etiological agent of two main diseases: a severe hematological disease with very poor prognosis called adult T-cell leukemia-lymphoma (ATL) [4], and an inflammatory syndrome involving the central nervous system named tropical spastic paraparesis/HTLV-1 associated myelopathy (TSP/HAM) [5]. The impact on health however is broader with multiple disease associations reported and an unexplained increase in mortality rates [6].

Despite low genetic diversity, HTLV-1 strains have been organized into several genotypes and subgroups [2]. There are 7 HTLV-1 genotypes (a to g), which often segregate according to the geographical origin of the infected individuals [7]. The “Cosmopolitan” a-genotype is distributed worldwide, while the other genotypes are geographically restricted: genotype c in Australo-Melanesia, and genotypes b and d to g in Central Africa. Within the a-genotype, several molecular clades have been defined: in Africa, there are the transcontinental (TC) clade, the West African (a-WA) clade, the North African (a-NA) clade, and the Senegalese (a-Sen) clade [7].

Purportedly, the major evolution mechanism for HTLV-1 is genetic drift. Point mutations are accumulated either during primary infection – with the usage of the viral reverse transcriptase (RT), which is error prone (with 7E-6 mutation/site/replication cycle) [8,9] –, or during clonal expansion of infected cells [10]. Overall HTLV-1 is a very stable virus with a mutation rate comprised between E-6 and E-7 substitution/site/year [11–13].

In 2014, we first suggested that recombination could also be at play in HTLV-1 evolution. Indeed, we demonstrated that HTLV-1 strains present in North Africa (a-NA clade) had originated from a recombination event between strains from the Senegalese (a-Sen) and the West African (a-WA) subgroups [14]. In order to better characterize these recombinant strains and their distribution in northwestern Africa, we sequenced (partially or entirely) a new series of 52 HTLV-1 strains from 13 North and West African countries and 4 strains from members of the Noir Marron community in French Guiana, who are descendent from African slaves [15]. Phylogenetic analyses of these new strains demonstrate the existence of at least 2 genotypes that have arisen from recombination events in this geographic area.

## Materials and methods

### Sample collection and ethics statement

**Figure 1.**
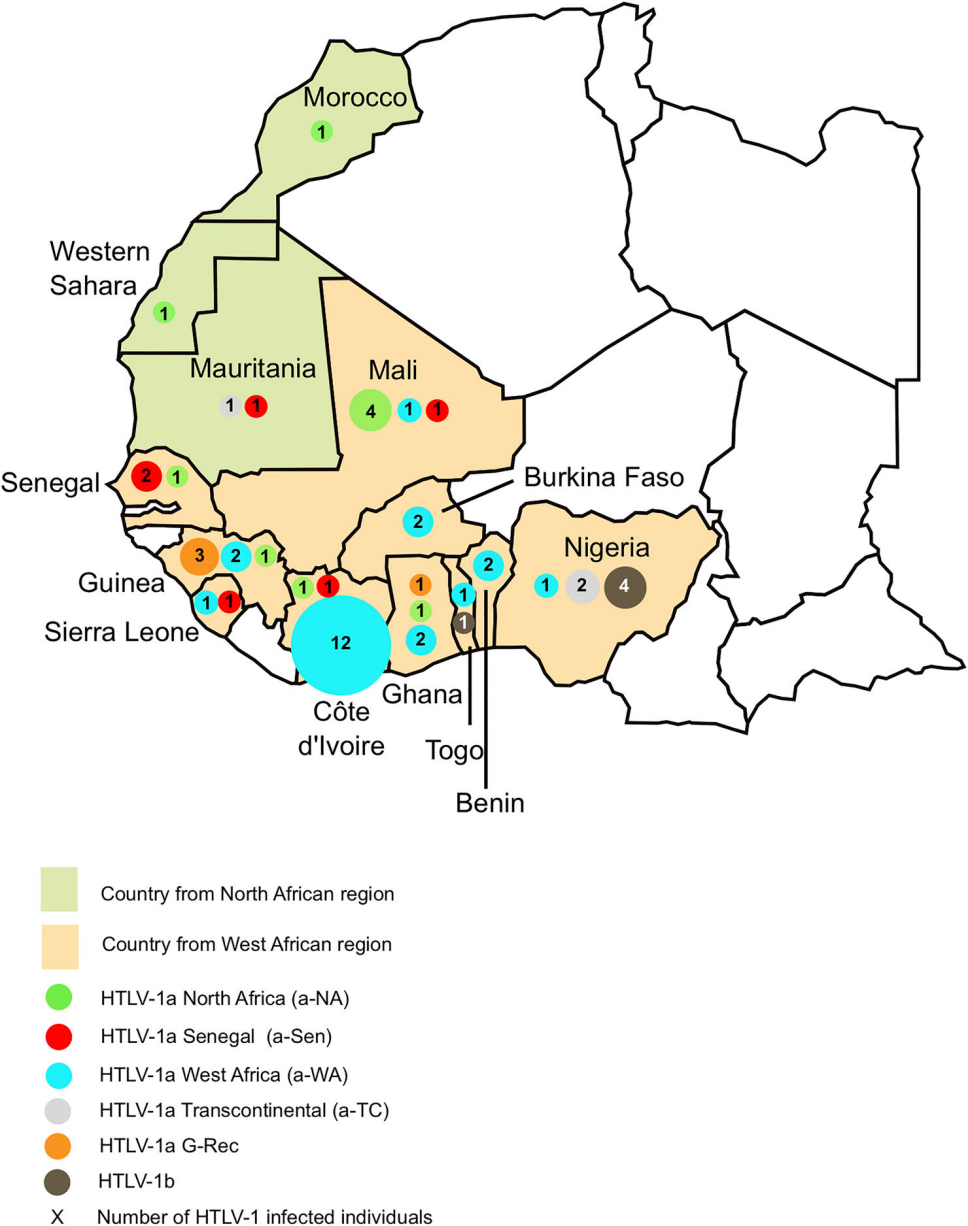
Geographical distribution of HTLV-1 strains in North and West Africa. The 52 HTLV-1 strains characterized were from Morocco (1), West Sahara (1), Mauritania (2), Mali (6), Senegal (3), Guinea (6), Sierra Leone (2), Côte d’Ivoire (14), Burkina Faso (2), Ghana (4), Togo (2), Benin (2), and Nigeria (7). The four strains from French Guiana are not represented on this map.

The studied samples were obtained from HTLV-1 infected individuals originating from different North and West African countries (Figure 1) and presenting various associated clinical conditions: ATL, TSP/HAM and asymptomatic carriers (Table 1). All these samples were collected in several hospitals that detect and monitor in- and outpatients infected with HTLV-1 in France, the United Kingdom and Spain. Samples obtained from descendants of African slaves called Noir Marron, and who escaped from Dutch plantations in the eighteenth century in Surinam (PH1049/PH1209/PH1211/PH1503), were also added to this series [15]. Samples were obtained according to French laws and regulations (Articles L.1211 and L.1243-3 from Code de la Santé Publique) in the context of a Biomedical Research Program approved by the Committee for the Protection of Persons, Ile-de-France II, Paris (2012-10-04 SC). The human sample collection has been declared to the Ministère de l’Enseignement et de la Recherche (2010 DC-1197). In the UK, samples were donated for research to the Communicable Diseases Group Tissue Bank, (ethics approval reference 15/SC/0089), following written informed consent. Samples collected in Spain belong to the repository of specimens from the Spanish HTLV Network, which is held at the Puerta de Hierro Research Institute in Madrid. Further data from this biological collection have been reported elsewhere [16]. All individuals have given their informed consent.

**Table 1.** Epidemiological data and clinical status of 56 HTLV-1-infected individuals of African descent for whom a 522-bp fragment of HTLV-1 gp21 and a 757-bp (complete) or a 402-bp (incomplete) fragment of the LTR region were amplified, sequenced and registered in GenBank.

Region of origin	ID	Sex	Age	Clinical status	Country of origin	PCR Env	PCR LTRg	PCR tLTR	HTLV-1 subtype / Subgroup from LTR analysis	Env Accession number	LTR Accession number
North Africa	KIN	F	44	Acute ATL	Morocco	+	+	+	a-NA	MN531973	MN532029
	Sah1454	F	32	AC	Western Sahara	-	+	NA	a-NA	-	MN532080
	PH1560	F	29	AC	Mauritania	+	+	+	a-TC	MN531975	MN532031
	Mau1371	M	51	TSP/HAM	Mauritania	+	+	NA	a-Sen	MN531976	MN532081
West Africa	COU	F	53	Acute ATL	Mali	+	+	+	a-NA	MN531977	MN532032
	KON.F	M	58	Chronic ATL	Mali	+	+	+	a-NA	MN531978	MN532033
	SIS	M	62	Acute ATL	Mali	+	+	+	a-NA	MN531979	MN532034
	KON.M	M	70	Lymphoma ATL	Mali	+	+	+	a-WA	MN531980	MN532035
	DIA.K	F	36	Acute ATL	Mali	+	+	+	a-Sen	MN531981	MN532036
	PH1620	F	32	TSP/HAM	Mali	+	+	+	a-NA	MN531982	MN532037
	PH1635	M	79	Neurological disorders	Senegal	+	+	+	a-Sen	MN531983	MN532038
	COL	M	39	Smouldering ATL	Senegal	+	+	+	a-Sen	MN531984	MN532039
	Sen68	M	50	AC	Senegal	+	+	+	a-NA	MN531985	MN532082
	PH1510	M	18	AC	Guinea	+	+	+	a-G-Rec	MN531986	MN532040
	BAL4	M	41	Chronic ATL	Guinea	+	+	+	a-NA	MN531987	MN532041
	PH1511*	F	52	AC	Guinea	+	+	+	a-G-Rec	MN531988	MN532042
	Gui107	M	52	AC	Guinea	+	+	-	a-WA	MN531989	MN532083
	PH541**	F	21	ATL	Guinea	+	+	+	a-G-Rec	MN531990	MN532043
	CON.B	F	32	Lymphoma ATL	Guinea	+	+	+	a-WA	MN531991	MN532044
	SL60	F	63	AC	Sierra Leone	+	+	+	a-WA	MN531992	MN532045
	SL34	F	77	AC	Sierra Leone	+	+	+	a-Sen	MN531993	MN532046
	KOU.Y	M	41	Lymphoma ATL	Côte d'Ivoire	+	+	+	a-WA	MN531994	MN532047
	DJO.K	F	32	AC	Côte d'Ivoire	+	+	+	a-WA	MN531995	MN532048
	IC1682	M	37	ATL	Côte d'Ivoire	+	+	+	a-WA	MN531996	MN532049
	DAF	M	62	Acute ATL	Côte d'Ivoire	+	+	+	a-WA	MN531997	MN532050
	DIA.H	F	40	TSP/HAM	Côte d'Ivoire	+	+	+	a-Sen	MN531998	MN532051
	GBA	M	51	Chronic ATL	Côte d'Ivoire	+	+	+	a-WA	MN531999	MN532052
	GNA6	F	57	Chronic ATL	Côte d'Ivoire	+	+	+	a-WA	MN532000	MN532053
	GNA7	M	51	Lymphoma ATL	Côte d'Ivoire	+	+	+	a-WA	MN532001	MN532054
	KEI	F	33	Lymphoma ATL	Côte d'Ivoire	+	+	+	a-NA	MN532002	MN532055
	NGO	F	42	Chronic ATL	Côte d'Ivoire	+	+	+	a-WA	MN532003	MN532056
	SAK	M	39	Smouldering ATL	Côte d'Ivoire	+	+	+	a-WA	MN532004	MN532057
	PH1605	F	43	AC	Côte d'Ivoire	+	+	+	a-WA	MN532005	MN532058
	PH1642	F	48	AC	Côte d'Ivoire	+	+	+	a-WA	MN532006	MN532059
	DRE	F	43	Acute ATL	Côte d'Ivoire	+	+	+	a-WA	MN532007	MN532060
	KOA	M	33	AC	Burkina Faso	+	+	+	a-WA	MN532008	MN532061
	KOA.H	F	35	Acute ATL	Burkina Faso	+	+	+	a-WA	MN532009	MN532062
	Gha92	F	52	AC	Ghana	+	+	+	a-WA	MN532010	MN532063
	KWA	M	61	Chronic ATL	Ghana	+	+	+	a-WA	MN532011	MN532064
	Gha1450	F	58	AC	Ghana	+	+	+	a-NA	MN532012	MN532065
	Gha1518	M	29	AC	Ghana	+	+	+	a-G-Rec	MN532013	MN532084
	MOG.B	F	56	Lymphoma ATL	Togo	+	+	+	a-WA	MN532014	MN532066
	AHO.M	M	56	Acute ATL	Togo	+	+	+	b	MN532015	MN532067
	QUE.D	M	68	AC	Benin	+	+	+	a-WA	MN532016	MN532068
	HOU.Y	M	45	AC	Benin	+	+	+	a-WA	MN532017	MN532069
	IYA	M	42	Acute ATL	Nigeria	+	+	+	b	MN532018	MN532070
	DES.*P*	M	28	Lymphoma ATL	Nigeria	+	+	+	a-TC	MN532019	MN532071
	JAL.*P*	M	45	AC	Nigeria	+	+	+	b	MN532020	MN532072
	Nig23	F	46	AC	Nigeria	+	+	+	b	MN532021	MN532073
	Nig21	F	51	TSP/HAM	Nigeria	+	+	+	a-TC	MN532022	MN532074
	Nig64	F	72	TSP/HAM	Nigeria	+	+	+	a-WA	MN532023	MN532075
	Nig1388	F	38	AC	Nigeria	+	+	-	b	MN532024	MN532085
French Guiana^$^	PH1049	F	30	Acute ATL	French Guiana	+	+	+	a-WA	MN532025	MN532076
	PH1209*	M	45	Lymphoma ATL	French Guiana	+	+	+	a-WA	MN532026	MN532077
	PH1211	M	45	ATL	French Guiana	+	+	+	a-WA	MN532027	MN532078
	PH1503	M	60	Acute ATL	French Guiana	+	+	+	a-WA	MN532028	MN532079

NA=Data or DNA Not Available

F=Female

M=Male

+= PCR amplification

- = no PCR amplification

*: Complete sequence (≈ 9 000-bp)

**: Incomplete sequence (≈ 6 000-bp)

^$^: Noir Marron individuals originating from West Africa (Brucato *et al*., BMC Evol Biol., 2010)

a-NA, a-WA, a-Sen and a-TC, North African, West African, Senegalese, and Transcontinental clades of the HTLV-1a genotype

a-G-Rec: New recombinant strains from Guinea and Ghana of the HTLV-1a genotype

b: African HTLV-1b genotype

### PCR detection and generation of *Env* gene, LTR fragments and complete HTLV-1 genomes

For the purpose of this study, high molecular weight DNA was extracted from peripheral blood buffy coat using the QIAamp DNA blood minikit (Qiagen, Hilden, Germany) or was referred directly to us by the medical unit in charge of monitoring patients infected with HTLV-1.

Samples were first amplified by PCR using the “env” primers pair; Env11: 5’-TGGCACG TCCTRTACTCTCCCAAC-3’ and Env22: 5’-GGCGAGGTGGAGTCCTTGGAGGC-3’, which was designed to amplify 885-bp-long fragment of the *envelope* gene. From each sample, 250 ng of DNA was amplified under the following conditions: 98°C, 1 min; 40X (98°C, 5 s; 72°C, 20 s); 72°C, 1 min. Reaction tubes were prepared in a dedicated room outside the laboratory with a final volume of 50μl (DNA matrix, 250 ng); dNTP mix (Roche, Basel, Switzerland), 40 mM; 5X Phire II reaction buffer which contains 1.5 mM MgCl2 at final reaction concentration (Ozyme, Saint Quentin-en-Yvelines, France), 10 μl; Phire II hot start DNA polymerase (Ozyme), 2 U and 0.5 mM of each oligonucleotide primer (Eurofins MWG, Ebersberg, Germany) [17].

Then, complete LTR sequences were obtained through two series of PCR, generating LTR-gag (LTRg) and Tax-LTR (tLTR) fragments with the primers pairs; Enh280: 5’- TGACGACAA CCCCTCACCTCAA-3’ and 5PLTR: 5’- TCCCGGACGAGCCCCCAA-3’ plus 8200LA: 5’- CTCACACGGCCTCATACAGTACTC-3’ and Rev3: 5’-GGAGGCACCACAGGCGGGAGGCG-3’ respectively. The two LTRg and tLTR segments obtained overlap by 197 bp (Fig S1), so we can concatenate them to obtain the complete LTR.

Finally, to generate the full-length sequences, we amplified four different HTLV-1 proviral genomic regions: F1 (2,145-bp), LTR-gag (with primers Enh280 and R2380); F2 (2,771-bp), pro-pol (with primers F2279 and R5005); F3 (2,226-bp), pol-env (with primers F4583 and Env22); F4 (2,280-bp), tax-LTR (with primers F6501 and 3VLTRext). For an extensive description of the technique and primers used, please refer to the manuscript published by Cassar *et al*. [18].

Ten microlitres of each amplified DNA fragment was size fractionated by 1.5% agarose gel electrophoresis. Then, the PCR products (40 μl) were sent for purification and sequencing reactions to the MWG Platform at Cochin Hospital, Paris, France.

The Clustal W algorithm (Mac Vector 17.0.5 software, Oxford Molecular) was implemented to align forward and reverse sequences of each segment, in order to obtain the consensus sequence of interest.

### HTLV-1 phylogenies

Multiple sequence alignments were performed with the DAMBE program (v4.2.13) [19]. For the studies on the *env* segment, no gaps or stop codons were observed.

The most appropriate nucleotide substitution model was selected in the Modeltest v3.6 program [20], based on the Akaike information criterion (AIC). The best-fitting models were GTR-Γ and Tamura-Nei-Γ for the LTR region and *env* gene sequences, respectively. Phylogenetic reconstructions were conducted in PAUP* v4.0b10 using the neighbour joining method (NJ) with 1,000 bootstrap replicates performed to test the robustness of the tree topology. Phylogenetic topologies were also confirmed using the maximum likelihood method (PhyML, on the SEAVIEW program) [21], and robustness of the groups was estimated by approximate likelihood test (aLRT). Bayesian approaches were inferred with the MrBayes 3.2.7 program [22]. Bayesian phylogeny was performed based upon the GTR substitution model (nst = 6, ngammacat = 6, rated = invgamma). The MCMC (Markov Chain Monte Carlo) analysis was performed with 4 chains (nruns = 2, nchains = 4) with the temperature set to 0.1. The chains ran for 2,000,000 cycles. The first 25% were discarded for the analysis (to compile only convergent data). The chains converged as the ESS was higher than 1,000 and the PSRF+ was equal to 1.

### HTLV-1 recombinant search

The recombinant search and breakpoint detection were performed by boot scanning in Simplot v3.5.1 [23]. This program compares inferred clusters of sequences to each other. Phylogenetic relationships of these clusters are estimated for successive overlapping sub-regions. For analysis on the LTR, we used a 200-bp-long window and a 20-bp-long step while for analysis on the entire genome, a 800-bp-long window and a 80-bp-long step was applied. We used different sizes of windows due to the lower variability in the other regions than the LTR. Thus a lower window would not be informative for the entire genome and would generate background noise.

For each window, the bootstrap value of the query and the references are calculated (according to the Kimura two-parameter model with 1,000 replicates). Bootstrap values are then plotted along the genome on an *x*/*y* plot, so that *x* values reflect the genome position at the midpoint of the analyzed windows and *y* values reflect the bootstrap value calculated from the windows. The divergent Mel5 strain (c-genotype) was used as outlier.

## Results

### Series of HTLV-1 infected individuals

Fifty-six HTLV-1 infected adults (28 women and 28 men) of African descent were included in this study (Table 1). The average age was 47 years old and ranged from 18 to 79. Four individuals were from North Africa (Morocco, Western Sahara, and Mauritania), 48 from West Africa (Mali, Senegal, Guinea, Sierra Leone, Côte d’Ivoire, Burkina Faso, Ghana, Togo, Benin and Nigeria) and 4 from French Guiana (Figure 1). Although French Guiana does not belong to the African continent, the infected individuals belonged to the Noir Marron ethnic group, who are of African origin and therefore carry a genome of African ancestry [15].

The clinical status of these individuals was diverse (Table 1). In addition to 20 asymptomatic HTLV-1 carriers, there were 30 ATL cases of various clinical presentations– smoldering (2), chronic (6), acute (11), lymphoma (8) or untyped (3) – and 6 individuals with neurological disorders, including 5 TSP/HAM.

### A highly supported sub-clade emerges within the Northern African clade

Amplification of the HTLV-1 LTR region was tested for the 56 samples from HTLV-1 infected individuals. Complete LTR sequences were obtained from all samples except 4, for which amplification of only the LTRg fragment was achieved (Table 1).

Alignment of the complete LTR sequences generated for the 52 strains revealed no significant deletion or insertion in comparison to the HTLV-1 ATK-1 reference strain, with the exception of two strains from Guinea (PH1510 and PH1511) that exhibit a deletion of 6-nt (position 638–643). In addition, the GBA strain from Côte d’Ivoire has an insertion of 18-nt (position 21–38), similar to the previously published HHZ strain. Comparisons of the new characterized strains indicates that they are closely related to each other with a nucleotide similarity range from 96% to 100%.

**Figure 2.**
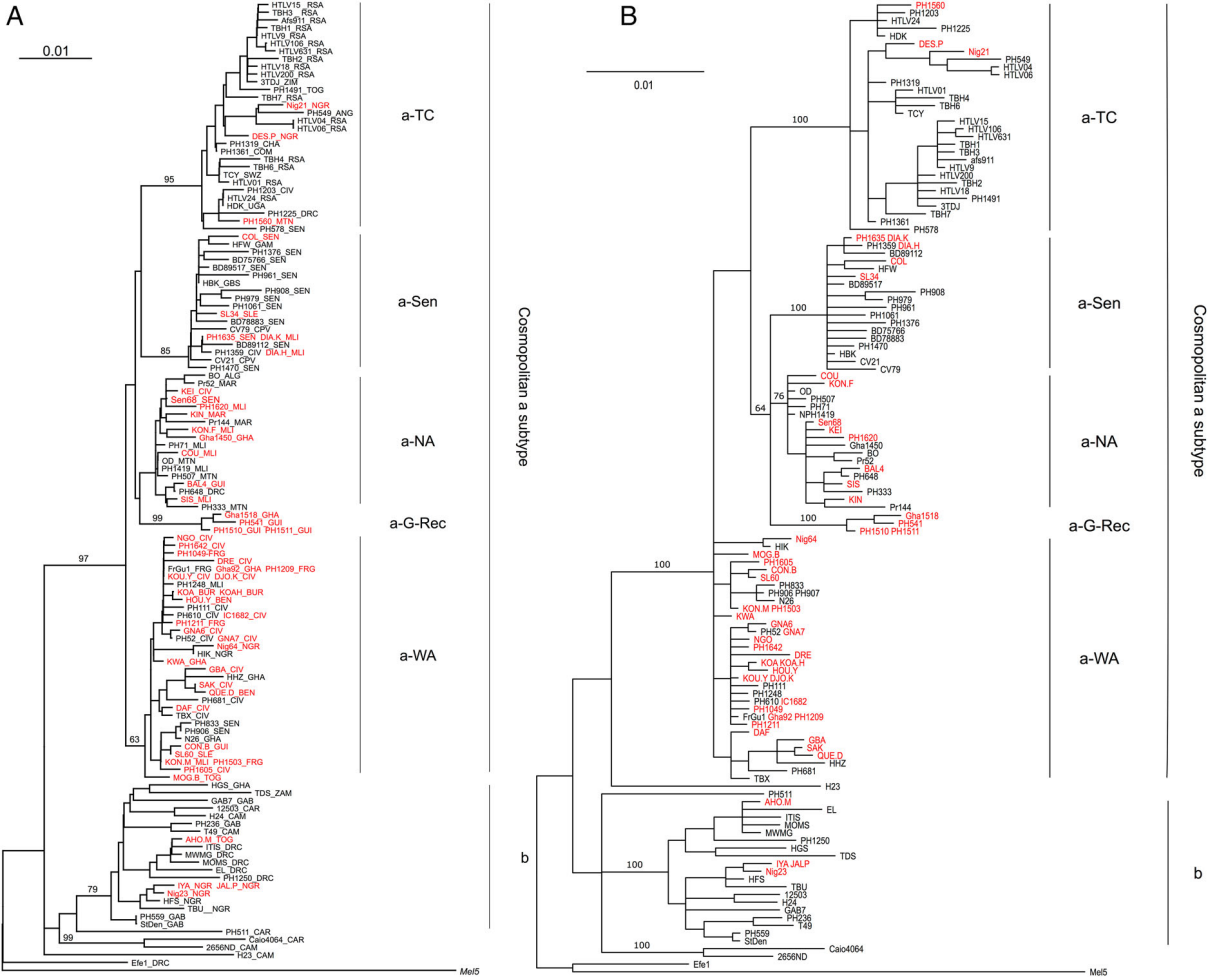
Phylogenetic analysis of African LTR sequences. A- Phylogenetic comparison was performed on 772-nucleotide-long LTR alignment of African isolates, including the 52 sequences generated in this study (in red). The Melanesian sequence *Mel5* was used as outgroup. The phylogenetic tree was derived by the neighbor- joining method using the GTR model (gamma = 0.5017). Horizontal branch lengths are drawn to scale, with the bar indicating 0.01 nucleotide replacement per site. Numbers on each node indicate the percentage of bootstrap samples (of 1,000 replicates) in which the cluster to the right is supported. Next to each sequence, three letters symbolize the country of origin of the infected individual (mostly IOC country codes): ALG - Algeria, ANG - Angola, BEN - Benin, BUR - Burkina Faso, CAM - Cameroon, CAR - Central African Republic, CHA - Chad, CIV - Côte d’Ivoire, COM - Comores, CPV - Cape Verde, DRC - Democratic Republic of Congo, FRG - French Guiana, GAB - Gabon, GAM - Gambia, GBS - Guinea-Bissau, GHA - Ghana, GUI - Guinea, MAR - Morocco, MLI - Mali, MTN - Mauritania, NGR - Nigeria, RSA – South Africa, SEN - Senegal, SLE - Sierra Leone, SWZ - Swaziland, TOG - Togo, UGA - Uganda, ZAM - Zambia, ZIM - Zimbabwe. B- Phylogenetic comparison was performed on 772-nucleotide-long LTR alignment of African isolates, including the 52 sequences generated in this study (in red). The Melanesian sequence *Mel5* was used as outgroup. The consensus phylogenetic tree was constructed using a Bayesian approach based upon the GTR substitution model. The MCMC analysis was performed with 4 chains that ran for 2,000,000 cycles. Horizontal branch lengths are drawn to scale, with the bar indicating 0.01 nucleotide replacement per site. Numbers on each node indicate the posterior probabilities of the branches (in percentage).

Phylogenetic analyses were performed on a 772-nt-long LTR alignment. The topologies of the phylogenetic tree were comparable for the NJ (Figure 2A) and the Bayesian approach (Figure 2B).

As expected, most (48/52 = 92%) of the new strains from Africa belong to the a-genotype (Figure 2). Only 4 strains (3 from Nigeria and one from Togo) belong to the b-genotype. Furthermore, as previously described [14], the large cosmopolitan HTLV-1a genotype can be subdivided into 4 subgroups: the transcontinental clade (a-TC), the Senegalese (a-Sen), the North African (a-NA), and the West African (a-WA) clades. As indicated by their names, these clades were globally related to a geographic region. As an example, 17/22 (77%) of the HTLV-1 strains from Côte d’Ivoire, Ghana, Togo and Benin belong to the a-WA subgroup. The subgroups were not related to disease; for example, strains from the a-NA subgroups were found in asymptomatic carriers, as well as ATL and HAM/TSP patients (Table 1).

Importantly, a phylogenetically supported subgroup composed of four sequences – originating from Guinea (PH541, PH1510, and PH1511) and Ghana (Gha1518) – emerged at the base of the a-NA monophyletic group. This group was provisionally named a-G-Rec (Figure 2A-B).

### A distinct recombination event leads to the new identified a-G-Rec clade

The a-NA group was previously identified as a recombinant group between a-WA and a-Sen [14]. Due to its close proximity to a-NA, we wondered whether the a-G-Rec group also emerged from a recombination event.

**Figure 3.**
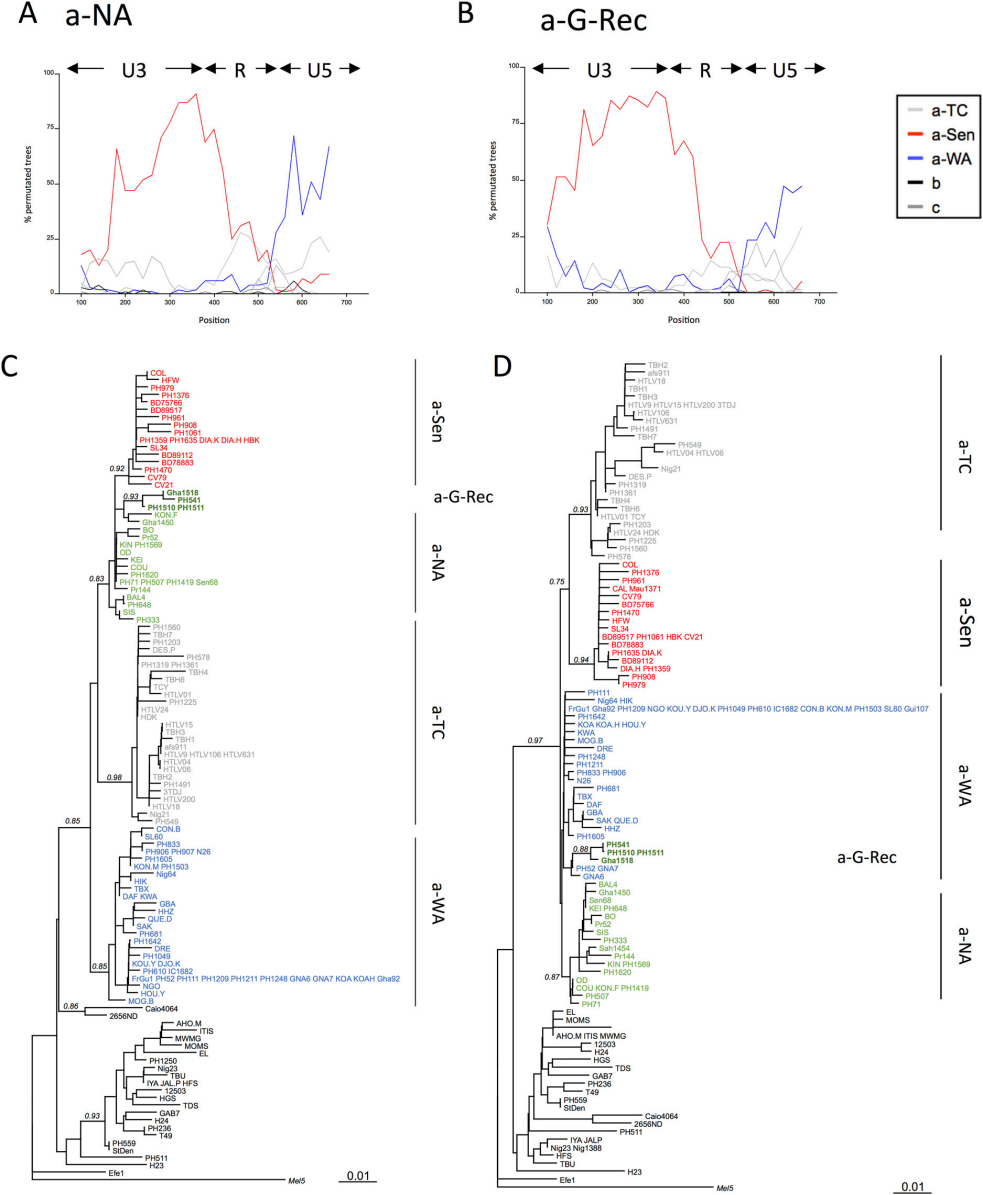
Boot-scanning for a-NA and a-G-Rec and Phylogenetic analysis of U3 and RU5 LTR segments. The a-NA (panel A) and the a-G-Rec (panel B) subgroups were compared by boot-scanning (Simplot program) to different clades (a-TC, a-WA, a-Sen, b, and c). The analysis used a 200- bp-long window and a 20 bp-long step, and the Kimura 2p model. The *x* values reflect the genome position at the midpoint of the analyzed windows, and the *y* values reflect the bootstrap value calculated from the windows (for 1,000 replicates). Phylogenetic trees corresponding to the first 371 nucleotides (Panel C) and the 401 last nucleotides (Panel D), respectively, were derived from the Maximum Likelihood method. Values correspond to the approximate likelihood-ratio test for each group. The groups of interest are coloured as follows: red, green, dark green, blue and grey sequences belong to a-Sen, a-NA, a-G-Rec, a-WA, and a-TC, respectively.

We studied the phylogenetic relationship of the different groups by the boot-scanning method. First, we confirmed that a-NA strains displayed a typical recombinant profile, with a U3 region closely related to a-Sen and a R-U5 region closely related to a-WA (Figure 3A). The newly identified a-G-Rec clade exhibited a similar profile (Figure 3B). When focusing on the nucleotide alignment, we found that a-G-Rec shared 4 specific nucleotides with a-Sen strains in the U3 region, and 4 specific nucleotides with a-WA strains in the R-U5 region, as we previously found for a-NA strains [14]. Together, this indicates that a-G-Rec strains likely derived from a recombination between a-WA and a-Sen, at the U3/R junction.

Separate analysis of the U3 and R-U5 segments of the LTR supported the mosaic profile hypothesis of the LTR of a-NA strains: on the U3 region, a-NA strains segregate with a-Sen strains, and on the R-U5 region, a-NA strain are closer to a-WA strains (Figure 3C-D). On the U3 segment, the a-G-Rec strains were found among a-NA strains; in contrast, on the R-U5 segment, the 4 a-G-Rec sequences formed a clade among a-WA but separate from a-NA (Figure 3C-D).

The separation between a-NA and a-G-Rec in the R-U5 segment may be due to specific mutations that have accumulated after the recombination event, or due to the fact that the parental a-WA strain was distinct for a-NA and a-G-Rec. In the latter hypothesis, a-G-Rec would have originated from a distinct recombination event.

### a-G-Rec and a-NA clade displays a mosaic profile with several recombination break points

We have previously suggested that a-NA strains were derived from a recombination that occurred during the first polymerase jump during reverse transcription (RT) [14]. In this hypothesis, the phylogeny from the gp21 *env* gene fragment should mirror the U3 phylogeny. A fragment of 885-bp-length of the *env* gene was thus obtained for all but one sample (Sah1454) by PCR amplification. Alignment of the gp21 *env* fragments for the newly obtained 55 sequences did not reveal any deletion or insertion or the presence of stop codon.

**Figure 4.**
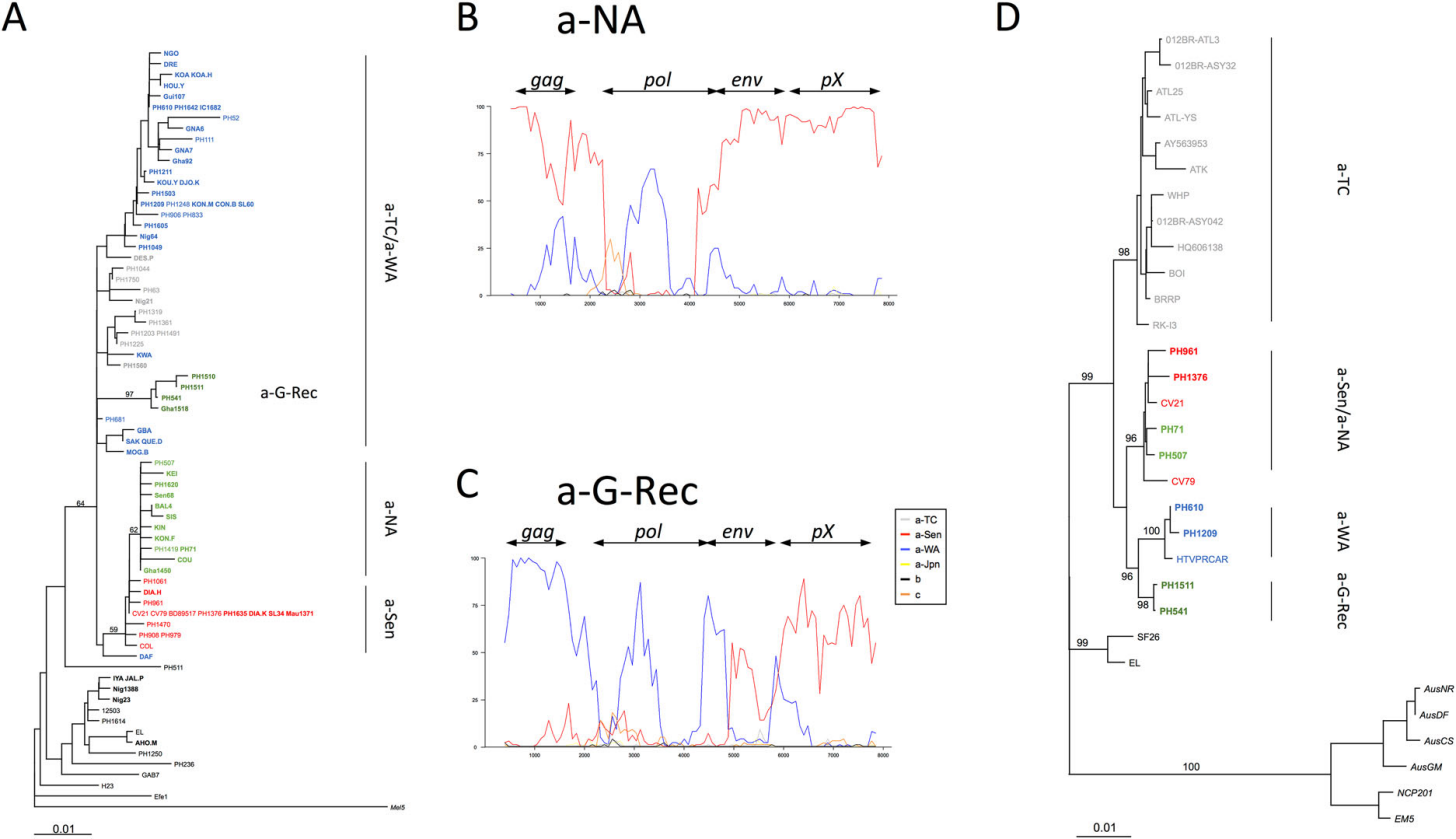
Evidencing the mosaic profile of a-NA and a-G-Rec and Phylogenetic analyses of *env* and *gag* genes. A- Phylogenetic comparison was performed on 522-nucleotide-long *env* gene fragments of African isolates. The Melanesian sequence *Mel5* was used as outgroup. The phylogenetic tree was derived by the Neighbor-Joining method using the Tamura Nei model (gamma = 0.3043). Horizontal branch lengths are drawn to scale, with the bar indicating 0.01 nucleotide replacement per site. Numbers on each node indicate the percentage of bootstrap samples (of 1,000) in which the cluster to the right is supported. Phylogenetic topologies were similar using different methods, i.e. Maximum likelihood and a Bayesian approach (data not shown). B-C- The a-NA (Panel A) and the a-G-Rec (Panel B) subgroups were compared by boot- scanning (Simplot program) to different clades (a-TC, a-Jap, a-WA, a-Sen, b, and c). The analysis used a 800-bp-long window and a 80-bp-long step, and the Kimura 2p model. The *x* values reflect the genome position at the midpoint of the analyzed windows, and the *y* values reflect the bootstrap value calculated from the windows (for 1,000 replicates). D- Phylogenetic comparison was performed on 2,094-nucleotide-long *gag* fragments (obtained from complete genomes). Six Australo-Melanesian HTLV-1c complete sequences were used as outgroup. The phylogenetic tree was derived by the neighbor-joining method using the Tamura Nei model (gamma = 0.8793; i = 0,5391). Horizontal branch lengths are drawn to scale, with the bar indicating 0.01 nucleotide replacement per site. Numbers on each node indicate the percentage of bootstrap samples (of 1,000) in which the cluster to the right is supported. Phylogenetic topologies were similar using different methods, i.e. Maximum likelihood and a Bayesian approach (data not shown). The groups of interest are coloured as follows: red, green, dark green, blue, and grey sequences belong to a-Sen, a-NA, a-G-Rec, a-WA, and a-TC respectively. New sequences are in bold.

Phylogenetic analyses were then performed on a 522-bp-long env segment (Figure 4A). As previously found [14], the a-NA strains and a-Sen strains formed a monophyletic group on the *env* fragment. Intriguingly, a-G-Rec strains did not branch in this group. Instead it formed a group within the a-TC/a-WA paraphyletic group (Figure 4A).

In order to better understand this striking observation, we needed complete genome sequences for the different subgroups. We generated 7 complete genome sequences: 2 from the a-NA subgroup, 2 from the a-WA subgroup, 2 from the a-Sen subgroup and 1 for the a-G-Rec subgroup (Table 2). The complete sequences were 9037 nt long, except PH1511 which was shorter due to a 6-nt long deletion in the LTR, as previously mentioned. *In silico* analysis showed that the different viral ORFs were present: the canonical *gag*, *pol*, *env* genes, the regulatory *tax* and *hbz* genes, and the accessory genes (*p12, p13, p30*). Furthermore, the splicing sites, as defined by Ciminale *et al.* [24], were conserved as well. Therefore these viruses seemed functional. Additionally, we generated a 6649-nt long sequence for the a-G-Rec PH541 strain; the pX region of this strain could not be sequenced.

**Table 2.** Epidemiological data and clinical status of 7 HTLV-1-infected individuals of African descent for whom the full-length HTLV-1 genome sequence was amplified, sequenced and registered in GenBank. An additional HTLV-1 genome sequence (PH541) was partially characterized.

ID	Country of Origin	Sex	Age	Clinical Status	Genotype	Subtype / Subgroup	HTLV-1 Complete Sequence Accession Number
PH610^$^	Côte d'Ivoire	M	59	ATL	HTLV-1a Cosmopolitan	HTLV-1a-WA	MN781152
PH1209	French Guiana	M	45	ATL	HTLV-1a Cosmopolitan	HTLV-1a-WA	MN781154
PH961^$^	Senegal	M	54	ATL	HTLV-1a Cosmopolitan	HTLV-1a-Sen	MN781153
PH1376	Senegal	M	29	AC	HTLV-1a Cosmopolitan	HTLV-1a-Sen	MN781155
PH71^$^	Mali	M	45	ATL	HTLV-1a Cosmopolitan	HTLV-1a-NA	MN781149
PH507^$^	Mauritania	M	25	ATL	HTLV-1a Cosmopolitan	HTLV-1a-NA	MN781150
PH541*	Guinea	M	21	ATL	HTLV-1a Cosmopolitan	HTLV-1a-G-Rec	MN781151
PH1511	Guinea	F	52	AC	HTLV-1a Cosmopolitan	HTLV-1a-G-Rec	MN781156

F=Female

M=Male

*: Incomplete sequence (≈ 6 600-bp)

^$^: Strains previously partially characterized in Env and LTR regions (Desrames *et al*., J. Virol, [14])

ATL=Adult T-cell Leukemia/Lymphoma

AC=HTLV-1 Asymptomatic Carrier

NA=Data Not Available

a-WA, a-Sen, a-NA and a-G-Rec, West African, Senegalese, North African and Guinea/Ghana Recombinant clades of the HTLV-1a Cosmopolitan genotype

We performed boot-scan analysis using HTLV-1 complete genomes available on GenBank. Such analysis revealed mosaic profiles for both a-NA and a-G-Rec genotypes (Figure 4B-C). a-NA was closely related to the a-Sen strain throughout the complete genome, but in a central portion of *pol*, where it is phylogenetically closer to a-WA strains (Figure 4B). In contrast, the 5’ region of a-G-Rec strains segregates with a-WA strains, while the 3’ region of the a-G-Rec genomes is closer to a-Sen strains (Figure 4C). The breaking points fall within the *env* gene. The differences between a-NA and a-G-Rec validated the striking observations on the *env* gene (Figure 4A). Moreover, in accordance with the boot-scan analysis, phylogenetic analysis on the *gag* gene revealed that a-NA strains belong to the a-Sen clade, and a-G-Rec strains form a monophyletic group with a-WA strains (Figure 4D).

We here demonstrate that a-NA and a-G-Rec strains have a mosaic profile with genetic segments deriving from either a-WA or a-Sen strains; as the mosaicism is distinct, it strongly suggest that a-NA and a-G-Rec have derived from distinct event.

## Discussion

The results we obtained in this study: 1) Confirm the geographical distribution of HTLV-1 clades in North and West Africa, 2) Confirm that the a-NA clade derives from a recombination event, and find a-NA strains in many parts of North and West Africa, 3) Identify a new genetic clade, named a-G-Rec, which arose from a distinct recombination event also between strains from the a-Sen and a-WA clades, 4) Show, based on the analyses of complete sequences, that a-NA and a-G-Rec strains depict a mosaic profile with genetic segments derived from either a-WA or a-Sen strains, suggesting that recombination does not only occur in the LTR but also in other regions (*env* and *pol*) of the genome. Both a-NA-Rec and a-G-Rec present a recombination break point at the same position of the LTR, i.e. the U3/R-U5 junctions. This point corresponds to the requisite change of RNA template during early reverse transcription (RT). As this first jump of RT is required to generate the LTR, this would explain why this particular junction seems to be a hotspot for recombination. Likewise, LTR was also previously reported as a recombination hotspot in HIV-1 [25]. Moreover, when analyzing the whole genome, we found that a-NA strains have a mosaic profile, with a central portion of the *pol* gene that resembles a-WA strains. Similarly, a-G-Rec strains also have a mosaic profile, with a second recombination point that occurred within the *env* gene. These recombination points may correspond to a template shifting during RT, which can occur thoughout the genome. For HIV, the recombination rate was estimated at 2.8 crossovers per genome per cycle [25].

In order to generate RT-related recombinations, two distinct RNA molecules have to be present in the same capsid. As a correlate, cells have to be infected with two viruses at a given time (either cells were infected simultaneously by two viruses, or sequentially through superinfection). However, multiple infection has not been observed in non leukemic primary cells until now [26]. This either means that multiple infection is rare, or that cells infected with many viruses may have a shorter lifespan, or that the cells infected with multiple viruses are not the circulatory T-cells that have been looked at.

Both a-NA and a-G-Rec have arisen from recombination between Senegalese (a-Sen) and West-African (a-WA) strains. One can wonder whether these two viruses are more likely to recombine, as other recombinants have not been evidenced so far. First, in order to identify a strain as a recombinant, there needs to be sufficient genetic diversity between the two parental strains. For instance, due to very low genetic diversity, recombination between two a-TC strains would be interpreted as simple point mutations. Recombination between a-WA and a-Sen strain is identified because there is just enough genetic diversity between these two clades. Second, there might be incompatibilities between some HTLV-1 genotypes. In central Africa, HTLV-1b, d, and a-TC strains coexist [14,27], but no recombinant between these genotypes has been reported yet. One possibility is that such recombinations exist but are to be reported. Another possibility is that hybrid strains cannot persist *in vivo*. We have recently found that HTLV-1b strains seem to lack accessory proteins P30 and P12 [28]. We hypothesized that either HTLV-1b express alternative accessory proteins, or HTLV-1b has accumulated throughout its genome compensatory mutations. One can postulate that the hybrid strain might not express proper accessory proteins and might not be viable.

The time and place where these recombination events occured remain unknown. For instance, we cannot determine whether recombination occurred where the derived strains are currently detected (North Africa and Guinea/Ghana for a-NA and a-G-Rec strains respectively), or whether it took place elsewhere (where a-Sen and a-WA strains are both present, i.e. in an area extending from Senegal to Mali and Ghana) and migrated secondarily to the regions where they are currently present.

Our work presents some limitations such the restricted sampling in some countries. It should be noted that most of our samples have been collected from African patients who had been medically attended in European hospitals (UK, Spain, and France). Thus, the samples obtained here are a direct illustration of migration between countries linked to the presence of former colonies in Western Africa (Nigeria, Ghana, and Sierra Leone for the United Kingdom; Western Sahara for Spain; Algeria, Morocco, Mauritania, Mali, Senegal, Guinea, Côte d’Ivoire, Burkina faso, Togo, and Benin for France).

Another limitation is the limited number of full-length sequences. Until now, most of the complete sequences available belonged to the HTLV-1a-TC genotype. In this study, we provide 7 new complete sequences from other clades (we report the first full-length sequences of HTLV-1a-G-Rec and HTLV-1a-NA). However, and due to the unavailability of most DNA samples based on their quality or quantity, we have not been able to characterize more complete sequences. Nevertheless, we were able to obtain at least two prototype sequences per identified clade. Nonetheless, obtaining complete sequences should be now on the standard objective in order to better identify potential recombinants.

In conclusion, while recombination has long been disregarded in HTLV-1 evolution, mosaic profiles can emerge and have been identified.
